# Biomedical Applications of Liquid Metal Nanoparticles: A Critical Review

**DOI:** 10.3390/bios10120196

**Published:** 2020-11-30

**Authors:** Haiyue Li, Ruirui Qiao, Thomas P. Davis, Shi-Yang Tang

**Affiliations:** 1 Department of Chemistry and Biochemistry, University of California, San Diego, CA 92093, USA; hal412@ucsd.edu; 2 ARC Centre of Excellence in Convergent Bio-Nano Science and Technology and Australian Institute for Bioengineering and Nanotechnology, The University of Queensland, Brisbane, QLD 4072, Australia; r.qiao@uq.edu.au; 3 Department of Electronic, Electrical and Systems Engineering, University of Birmingham, Edgbaston, Birmingham B15 2TT, UK

**Keywords:** liquid metal, nanoparticles, biomedical applications, EGaIn, Galinstan, gallium

## Abstract

This review is focused on the basic properties, production, functionalization, cytotoxicity, and biomedical applications of liquid metal nanoparticles (LMNPs), with a focus on particles of the size ranging from tens to hundreds of nanometers. Applications, including cancer therapy, medical imaging, and pathogen treatment are discussed. LMNPs share similar properties to other metals, such as photothermal conversion ability and a propensity to form surface oxides. Compared to many other metals, especially mercury, the cytotoxicity of gallium is low and is considered by many reports to be safe when applied in vivo. Recent advances in exploring different grafting molecules are reported herein, as surface functionalization is essential to enhance photothermal therapeutic effects of LMNPs or to facilitate drug delivery. This review also outlines properties of LMNPs that can be exploited in making medical imaging contrast agents, ion channel regulators, and anti-pathogenic agents. Finally, a foresight is offered, exemplifying underexplored knowledge and highlighting the research challenges faced by LMNP science and technology in expanding into applications potentially yielding clinical advances.

## 1. Introduction 

Gallium (Ga)-based liquid metal alloys, as metallic fluids at ambient temperature, exhibit fluidic flexibility in shape and size in addition to standard metallic properties, including high thermal and electrical conductivities, high densities, and the ability to respond to electric and magnetic fields [1]. When compared to mercury, the low cytotoxicity of Ga opens up new opportunities for liquid metal materials particularly in bio-related fields [2]. Most metallic elements (all alkali and alkaline earth, and most basic and transition metals) can dissolve in Ga to form alloys [3]. The two most commonly used Ga-based alloys are eutectic gallium indium (EGaIn, 75% Ga and 25% In by weight) [4] and eutectic gallium-indium-tin [5] (Galinstan, which has several composition ratios, typically 68% Ga, 22% In and 10% Sn by weight). EGaIn and Galinstan are notable for their low melting points, 15 and 11 °C, respectively. Unlike mercury (also liquid at room temperature), Ga-based alloys are more chemically reactive (as they instantly react with oxygen to form an oxide layer), but far less toxic and possess a negligible vapor pressure [1]. As commercial products, they are accessible to most labs, facilitating widespread research and application.

As bulk Ga-based alloys are processed into nanoparticles (NPs), they acquire many unique but useful properties that are beneficial in biomedical applications, as listed below:

(1) Surface oxide layer: One unique property of Ga-based alloys is the existence of a thin layer of oxide that effectively yields a core-shell structure in NPs, under normal processing conditions [6]. The oxide skin forms rapidly when the inner Ga core is exposed to oxygen. The formation of an oxide layer is beneficial in the production of LMNPs as it helps stabilize the surface to provide a barrier to particle–particle interactions; this becomes particularly important when small NPs are required.
(2) A grafting platform: The oxide layer yields a shell that can be exploited to attach or anchor surface functionality—this is vital for biomedical applications where stealth and targeting are widely required for effective application.
(3) Photothermal conversion ability: LMNPs of Ga alloys possess a relatively good photothermal conversion efficiency (52%) and a wide range of light absorbance (near-infrared light (NIR), 650–1500 nm) [7], both key features for applications in photothermal therapy.
(4) Response to remote signals: LMNPs undergo chemical (leading to morphological) changes upon the application of an electromagnetic, acoustic, or alternating electric field, potentially providing a trigger for the release of loaded drugs [7,8,9,10,11,12,13,14].
(5) Cancer suppressor: Ga has been used for different clinical applications in forms of compound and ion due to its ability to modify structures of DNA, inhibit activities of enzymes, modulate protein synthesis, and alter the permeability of plasma membrane. For example, Ga^3+^ ions have shown theragnostic effects for hypercalcemia, and therapeutic effects against non-Hodgkin’s lymphoma and bladder cancer [15,16].

**Figure 1:**
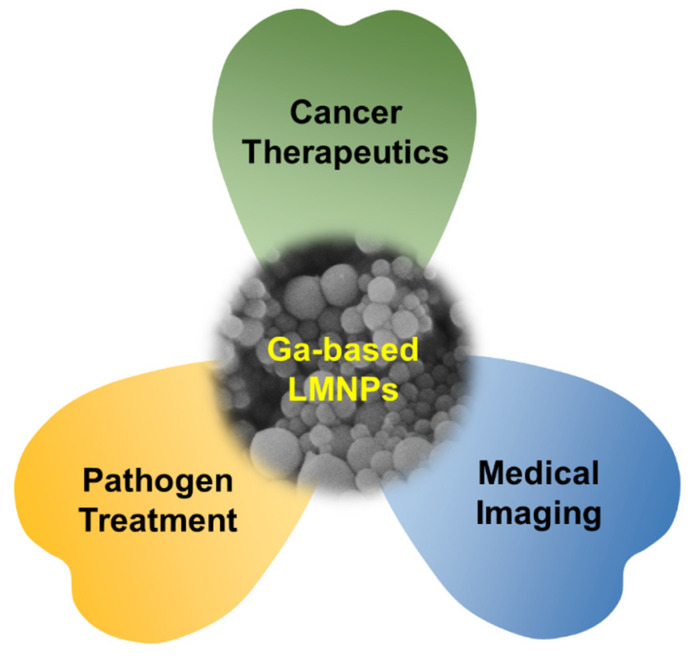
Biomedical applications enabled by Ga-based liquid metal nanoparticles (LMNPs).

This review seeks to highlight exciting advances in the use of the Ga-based LMNPs for biomedical applications, including cancer therapy, medical imaging, and pathogen treatment (Figure 1). The paper will focus on liquid metal particles with a size range of tens to hundreds of nanometers, and we use the generic abbreviation LMNPs throughout this manuscript. Different aspects of liquid metals, such as their applications in microfluidics, soft electronics, smart materials, forming composites, and injectable biomedical technologies, have been reviewed elsewhere [17,18,19,20,21,22,23]. This review is therefore focused on describing the strategies for the production and biofunctionalization of LNMPs and the presentation of how these NPs can be used in various biomedical applications. Finally, the review offers a perspective on the opportunities and challenges for LMNPs in future biomedical applications as research progresses towards clinical outcomes.

## 2. Production of LMNPs 

**Figure 2:**
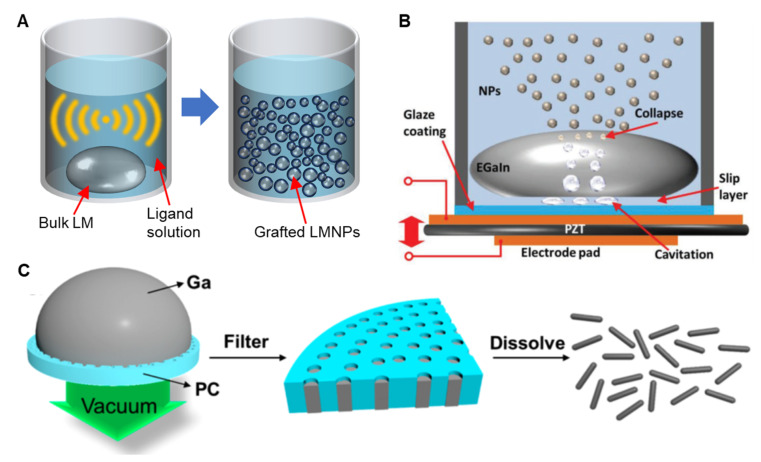
Methods developed for producing biofunctional LMNPs. (**A**) Schematic showing sonicating bulk liquid metal within a solution containing grafting molecules for one-step production of biofunctional LMNPS. (**B**) Schematic illustrating the liquid-based nebulization method for producing LMNPs. Reproduced with permission from Wiley [30]. (**C**) Scheme showing the preparation of rod-shaped LMNPs through the pressure-filter-template method. Reproduced with permission from ACS [13].

The manufacturing process of LMNPs—sonication, the most prevalent top-down synthesis method, generally takes from several minutes to hours—is less time consuming and simpler when compared to the equivalent processes required for synthesizing rigid NPs. While the production and assembly of grafting molecules onto other solid metal particles requires time consuming and complex techniques, co-sonicating bulk liquid metal within a solution containing grafting molecules enables a one-step process for the concurrent production and functionalization of LMNPs [24]. During sonication, the bulk metal is sheared into smaller pieces and eventually micro or nano-size particles (Figure 2A). The size distribution of obtained particles can be controlled by regulating the power and sonication time. Generally, a longer sonication time leads to a smaller average diameter and a narrower size distribution of LMNPs [25,26].

As metal particles form, the surfaces oxidize almost immediately, providing a barrier to particle coarsening or remerging, minimizing any reversal of the particle formation process. It is worth noting that, since the self-limiting layer is an oxide skin, any change in the environment’s pH that inhibits oxide formation could lead to the merging of particles [27]. The rapid and highly localized heat induced by intense acoustic power can lead to undesirable side effects, such as the formation of oxide nanorods resulting from dealloying and over-oxidation [28]. Lowering the temperature of the suspension using an ice bath can prevent the formation of adverse side products. Alternatively, using a dynamic temperature control system incorporating Peltier cooler pads and a feedback control module can improve the production performance of LMNPs [29]. Maintaining a constant temperature results in a significant increase in particle concentration and a decrease in overall size. On the other hand, liquid-based nebulization technique shears the bulk liquid metal within a carrier fluid by collapsing cavities generated by an ultrasonic piezoelectric transducer (Figure 2B) [30]. The nebulization approach lacks the ability to control the size distribution of LMNPs. However, it generates a lower suspension temperature and has less reagent consumption. The pressure-template method is another approach to fabricate non-spherical LMNPs [13,31]. As the name suggests, this process works by pushing bulk liquid metal into a pre-made polymer template with the template subsequently removed by dissolution, the obtained LMNPs often end up needle shaped (Figure 2C). Moreover, Tevis et al. proposed an emulsion shearing method that could synthesize LMNPs with complex morphologies [32]. With this method, LMNPs are produced at room temperature in the presence of a carrier fluid using a high-speed rotary spear.

A bottom-up synthetic approach to LMNPs has also been adopted in a few studies, where atoms aggregate to build larger and more complex nanoscale structures. Thermal decomposition and physical deposition are the primary bottom-up methods. The synthesis of monodispersed Ga NPs was achieved by Yarema et al., exploiting the thermal decomposition of Ga alkylamides to yield near-monodispersed Ga NPs with accurate size control [33]. Smaller LMNPs were collected almost immediately and remained stable for several months, while larger particles would slowly precipitate upon storage. Alternatively, depositing a physical vapor of liquid metal onto solid substrates via thermal evaporation can also produce surfactant-free polydispersed LMNPs in a dry environment [34]. Redispersing the produced NPs in a liquid environment has yet to be explored for particles produced in this way. Table 1 shows a comparison of the abovementioned top-down and bottom-up LMNP production methods.

## 3. Surface Functionalization of LMNPs 

**Figure 3:**
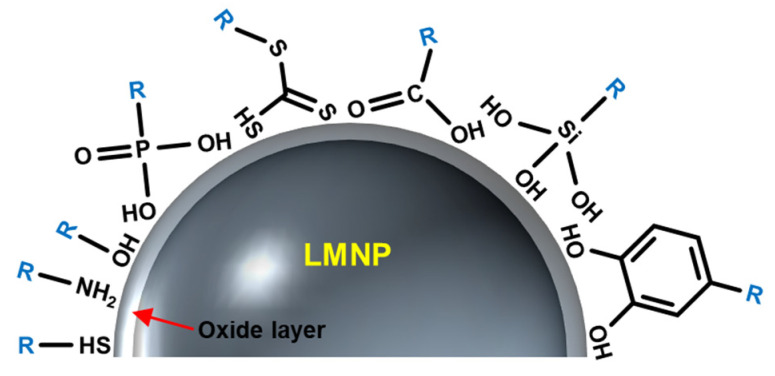
Molecules with different anchoring groups have been used for grafting the surface of LMNPs.

Surface grafting reactions are required to fully exploit the potential of LMNPs. The grafting process alters the surface properties of LMNPs to mediate aggregation, ligand exchange, release of Ga ions, and further oxidation of LMNPs in biological buffers, thereby increasing their colloidal stability. Previous efforts have attempted to use a range of macromolecules with different anchoring groups, such as thiol [24,27,35], catechol [36], phosphonic acid [29,37], trithiocarbonate [38], carboxylic acid [39,40], silane [41], and amine [7,28,42] to graft the NPs (Figure 3), but achieving long-term stability of LMNPs in biological buffers is an ongoing challenge.

Thiolation is a common grafting reaction utilized to functionalize LMNPs. It has been hypothesized that thiolated molecules inhibit the growth of the oxide layer [35]. A reasonable mechanism has been postulated concerning this finding, even though not fully validated through experiments; for group III metals, such as Ga, indium (In), and aluminum undergoing surface oxidation, adsorption of oxygen onto the particle surface creates a new and transitional surface state. Adsorbed molecules such as thiols can compete with the oxygen adsorption process, thereby suppressing the oxide layer growth.

Phosphonic acid, as an anchoring group, has been shown to facilitate the stability of EGaIn NPs colloids at room temperature [29,37]. In contrast to thiolation, where reversible physisorption is the dominant process, phosphonic acids undergo a series of condensation reactions to form covalent bonds. Phosphonic acid has been applied in the production of LMNPs with an oxide surface to modify the surface wettability and electronic work functions. Moreover, a brushed polyethylene glycol (bPEG) polymer with multiple phosphonic acid anchoring groups was designed to optimize graft stability to LMNPs, thereby preventing the attached macromolecule from surface dissociation resulting from any potential for competitive ligand exchange in biological buffers [29].

Poly(1-octadecene-*alt*-maleic anhydride) (POMA) is a macromolecule which can provide a hydrophobic layer to insulate LMNPs from chemical reactions, while hydrolyzed maleic anhydride yield hydroxyl groups that can sterically stabilize the NPs [39]. With POMA modification, the storage period of LMNPs can be increased up to 60 days in biological buffers. In addition, a silanization strategy has also been demonstrated for coating LMNPs [41]; hydroxyl groups on Ga oxide, can be reacted with alkoxysilane ligands to form silane linkages between the silicon center of the alkoxysilane and the particle surface. With this modification, LMNPs can be used as stretchable conductors at a scale smaller than otherwise possible. Through these anchoring groups, various functional biomacromolecules can be conjugated to the surface of LMNPs, enabling various biomedical applications, as exemplified in the following sections.

## 4. Toxicity Evaluation

The toxicity of metals and their ions can be quantified based on their cellular uptake. The pharmacokinetic parameter is defined as the fraction of the drug reaching systemic circulation following administration. The measurement describes how likely the metal ions bound to biomacromolecules (via a protein corona) enter cells through cell membranes [43]. Considering the complexity and diversity of the human body, each time LMNPs with new anchoring groups or assistive structures are designed, individual cytotoxicity evaluations must be conducted based on the specific pH, temperature, and degradation path.

When EGaIn is placed in an aqueous environment, water reacts with the LMNPS surface, leading to the formation of gallium oxide hydroxide (GaOOH) crystallites [44,45]. Ga ion is released by the below mechanism.

$$GaOOH+H_{2}O\;↔Ga^{3+}+OH^{-}$$

It is obvious that Ga ions are released as a result of hydrolyzation of the surface oxide layer. Therefore, the dominant species found in water is Ga ion, but rarely In ion, which is hidden in the core. Kim et al. determined that there is a positive, linear relationship between the surface area of EGaIn and ion release via experimental studies [46]. With the presence of physical disturbance, such as sonication, the bulk metal is broken into smaller fractions, which increases the surface oxide area and therefore the concentrations of both Ga ion and In ions. In addition, during sonication, the extreme and localized heat causes reactive radicals to form leading to further oxidation, thus releasing even more metal ions.

**Figure 4:**
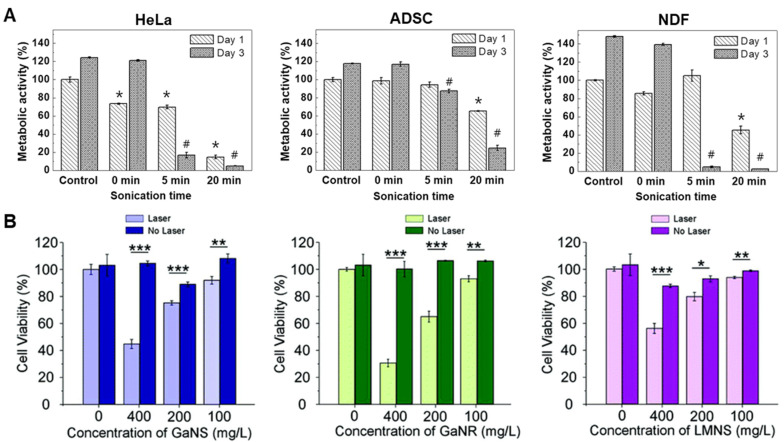
Cytotoxicity evaluation of LMNPs. (**A**) Metabolic activity of HeLa cells, ADSCs, and NDFs exposure to EGaIn releasates at different sonication time. * and # indicate significant differences as compared with day 1 and 3 controls (cells with no EGaIn releasates), respectively (*p* < 0.05). Reproduced with permission from ACS [46]. (**B**) Cell viability of MDA-MB-231 cells cultured with GaNS, GaNR and LMNS at a concentration of 400 mg/L. The external laser used was 1.5 W for 4 min. * *p* < 0.05, ** *p* < 0.01, and *** *p* < 0.001 compared with the control group. Reproduced with permission from RSC [12].

To further evaluate the cytotoxicity of EGaIn and its released ions, in vitro experiments have been conducted by culturing cells of different types in media, including ion releasates [46]. Human cell lines of HeLa, adipose-derived stem cells (ADSCs), and neonatal dermal fibroblasts (NDFs) were cultured with the addition of EGaIn releasates that were prepared by incubating EGaIn for 24 h, with a sonication time of 0, 5, and 20 min. Based on data provided (Figure 4A) [46], it can be concluded that there is negligible cytotoxicity of EGaIn releaseats through natural dissolution and, therefore, EGaIn can be considered safe for a range of differnt cell types. In a following experiment the effect of relesate concentration was studied; after 20 min of sonication, the concentrations of Ga and In ions released by EGaIn increased to ~120 and ~240 μM, respectively. The metabolic activity for all three types of tested cells were significantly reduced, indicating the importance of concentration profiles. Based on this study, a concentration–safety line could be drawn, above which the Ga ions may become too concentrated to be considered safe. Therefore a cytotoxicity window needs to be established for every in vivo formulation to ensure safe application.

Tang et al. also examined the cytotoxicity of LMNPs grafted with different molecules on the MCF-7 cell line using the Alamar Blue assay [30]. The tested surface grafting molecules include brushed polyethylene glycol (bPEG, MW of 20 kDa), poly(methyl vinyl ether-*alt*-maleic anhydride) (PMVEMA, MW of 216 kDa), poly(styrene-*co*-maleic anhydride) (PSMA, MW of 224 kDa), and oleic acid (OA). The survival rates of MCF-7 cells under the effect of LMNPs of different surface grafting molecules were investigated, leading to the conclusion that, up to a certain particle concentration (0.1 mg/mL), LMNPs with all four types of surface grafting could be considered non-toxic, as the survival rates of MCF-7 cells are either close to or exceeding 100%. When the concentration of LMNPs reached 0.1 mg/mL, MCF-7 cultured with LMNPs, except for LMNPs with OA, showed a decrease in survival rate to ~85–90%, indicating a cut off point for cytotoxicity. Additional experiments are now required to probe not only concentration effects but also timelines, in association with standard in vivo characterization such as pharmacokinetics and biodistribution studies.

The cytotoxicity of LMNPs with different shapes and compositions was evaluated by Sun et al. [12] who tested Ga nanospheres (GaNS), Ga nanorods (GaNR), and Ga-In liquid metal alloy nanorods (LMNR) in MDA-MB-231 cells (Figure 4B). This experiment showed that, without an external laser stimulus, GaNS, GaNR, and LMNR could be considered non-toxic, even at a high concentration of 400 mg/L.

In addition to the toxicity of Ga ions, GaOOH NPs could also be a major toxin. The amount of GaOOH crystallite is directly proportional to the percentage of water in the processing solvent [44]. LMNPs can be stored in an organic solvent such as pure ethanol to avoid the formation of GaOOH. In hypoxic conditions, a large amount of GaOOH formed, followed by sonication. While in ambient conditions, GaOOH crystallites do not form until hours of incubation in water. The generation of reactive oxygen species (ROS) is thought to be linked to the shape of GaOOH particles, and the imbalance of ROS is potentially detrimental that can lead to accelerated cell death [47].

## 5. Biomedical Applications of LMNPs 

LMNPs possess a range of properties that make them attractive candidates as biomaterials. The oxide skin is a key feature of gallium-based alloys, both in bulk form and nano formulations. Upon exposure to ambient oxygen, a thin Ga_2_O_3_ film with a nominal thickness of ~0.7 nm instantly forms [48,49,50]. In non-vacuum settings, environmental perturbation would continue thickening the oxide layer to ~3 nm [51]. When bulk Ga-based liquid metals are broken into fractions or NPs, this oxide skin reforms immediately. Moreover, this oxide skin forming ability does not disappear upon alloying. This oxide skin functions to stabilize the metal particle and forms a core-shell structure. This oxide layer can be chemically removed by changing the environment pH to either acidic (pH < 3) or basic (pH > 11.5) at room temperature [52].

**Figure 5:**
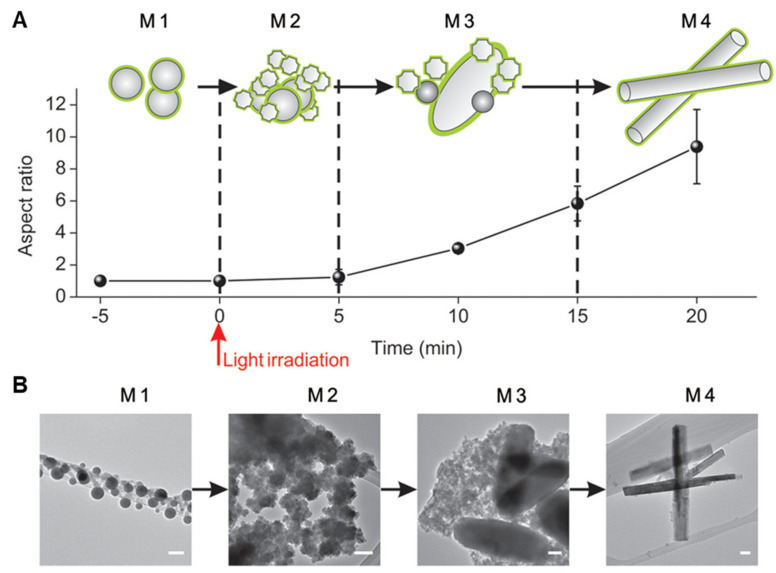
Shape transformation of graphene quantum dot (GQD)-coated LMNPs. (**A**) Schematic changes the four stages (M1, M2, M3, and M4) of LMNPs over light irradiation time. (**B**) Representative TEM images of the four transformation stages. Scale bars: 100 nm. Reproduced with permission from ACS [14].

Shape transformability is another defining property of LMNPs [7,14]. With proper external stimulation, such as heat or light irradiation, LMNPs in an aqueous environment undergo chemical oxidation and therefore, undergo dramatic morphological changes. Herein we discuss a typical example; Yue Lu et al. revealed in detail the nature of the shape transformation in LMNPs coated with graphene quantum dots (GQD) stimulated with 635 nm wavelength radiation [14]. The shape-changing process can be codified into four stages (named as M1, M2, M3 and M4), as shown in Figure 5. From M1 to M2 the nanofragments grow from spherical particles (M1). During the transition from M2 to M3, the particle resembles an oval-shaped sheet (M3). Following continuous light irradiation, the oval sheets morph into rod-like nanostructures. This experiment revealed that the morphological changes induced in LMNPs result from oxidation of Ga: energy dispersive spectroscopy mappings show the overlap of Ga and O signals that are measured during stages M2 to M4, in which the major shape transformation takes place. It is hypothesized that similar mechanisms govern the shape transformation of most LMNPs. An essential feature still under-investigated is an ability for both bulk and nano LM to undergo photothermal conversion processes.

### 5.1. Treatment against Cancer 

Morphological transformation properties make LMNPs attractive in applications such as cancer therapy. Applying Ga-based LMNPs to cancer therapeutics is a relatively new concept, with a few pioneering studies suggesting strong potential for future applications, as illustrated in the following examples.

**Figure 6:**
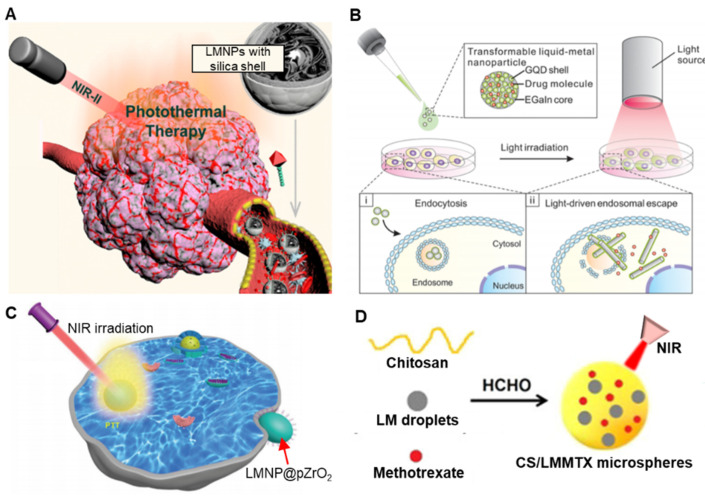
Different modification strategies of LMNPs for cancer treatment. (**A**) Demonstration of silica-coated LMNPs loaded with doxorubicin (DOX) for photothermal therapy (PTT). Reproduced with permission from ACS [54]. (**B**) Multi-functional GQD-coated LMNPs that can deliver DOX and disrupt the interior of cancer cells via shape transformation. Reproduced with permission from ACS [14]. (**C**) Demonstration of LMNPs-coated with ZrO_2_ for PTT. Reproduced with permission from RSC [58]. (**D**) Microspheres incorporating chitosan, Ga liquid metal, and methotrexate for PTT. Reproduced with permission from ACS [59].

Photothermal therapy (PTT) as a cancer therapeutic is an established field. Upon external stimuli, NIR-II irradiation for example, NPs of inert metals such as gold (Au) nanorods, can be excited to release heat, thereby killing any adjacent tumor cells [53]. The enhanced permeability and retention (EPR) effect can be exploited to concentrate nanoparticles in vivo at the site of leaky tumours. As LMNPs possess the ability to induce PTT, this has attracted a number of research groups to explore their potential. Zhu et al. designed LMNPs within an inorganic silica nanoshell, to achieve multiple therapeutic effects (Figure 6A) [54]. The silica coating (tetrathylorthosilicate) imbues the LMNPs with photostability by preventing further oxidation of liquid metals. In addition, the silica coating provided an extra layer to facilitate the LMNPs to be further conjugated with additional biomolecules via silane chemistry. The photothermal heating performance of silica coated LMNPs was evaluated using NIR irradiation at a wavelength of 1064 nm. Different powered densities and solution concentrations were tested to find the optimal photothermal conversion efficiency, which assess the capability of converting light energy to heat; this was calculated to be 22.3%, superior to bare liquid metal (14.12%), tin selenide (SnSe) nanorods (20.3%), Au nanorods (21%), or palladium nanosheets (20%).

Other groups have also proposed modified applications of LMNPs for PTT. Hu et al. proposed LMNPs with a mesoporous silica coating [55], and Wang et al. proposed LMNPs with a natural leukocyte membrane shell [31]. Wang et al. designed shape transformable LMNPs that combined PTT with photodynamic therapy (PDT) through light-fueled shape transformation to kill cancer cells [31]. Chechetka et al. grafted 1,2-distearoyl-*sn*-glycero-3-phosphoethanolamine-*N*-[amino(polyethylene glycol)-2,000] (DSPE-PEG_2000_-Amine) and 1,2-bis(10,12-tricosadiynoyl)-*sn*-glycero-3-phosphocholine (DC(8,9)PC) onto the surface of LMNPs [7]. The coated LMNPs exhibited enhanced water dispersibility. This design also yielded strong photothermal conversion performance; LMNPs have superior photothermal stability over an organic dye molecule (such as indocyanine green), the traditional reagents used in NIR photodynamic therapy. Zhang et al. synthesized LMNPs coated with tumor cell membrane, which could release heat under 808 nm irradiation, generating an immune response in that locality [56]. Antigen-presenting cells could be activated and elicit an anti-tumor response. Hu et al. constructed LMNPs coated with glucose oxidase (GOX) that functions as starvation therapy. The starvation effect also causes an increase in the flow rate of blood and could contribute positively to the effect of light-induced PTT [57].

The ability to load drugs to stable core-shell nanostructures is well established and the exploitation of LMNPs as delivery vectors has also been explored in a number of studies. The oxide layer on LMNPs can act as a scaffold or a storage layer that can dissolve and release any bound contents upon the application of an external stimulus, such as pH, NIR light, electric, magnetic, or acoustic field. Lu et al. loaded doxorubicin (DOX) into coated LMNPs [27]. Two ligands, thiolated (2-hydroxypropyl)-b-cyclodextrin (MUA-CD) and thiolated hyaluronic acid (m-HA) were used as capping agents during the production process. The cyclodextrin formed an inclusion complex to store and deliver DOX, and the m-HA moiety was exploited to target CD44 receptors, a typical overexpression at the tumor region. The same group also proposed GQD-coated LMNPs to deliver DOX using a light-induced transformation process (Figure 6B) [14].

The cytotoxicity of DOX-loaded LMNPs (LMNPs@DOX) was evaluated using HeLa cells. The half-maximal inhibitory concentration (IC_50_) towards HeLa cells for 24 h treatment was 0.81 and 0.21 mg/L, respectively, which proved that LMNPs@DOX were more effective therapeutics than free DOX solutions, whose IC_50_ was 1.33 mg/L. Additionally, it can be concluded that the cytotoxicity of LMNPs@DOX is time and concentration dependent. It is hypothesized that an acid-promoted release of DOX provided a higher cytotoxicity activity. Based on this finding, the experimental study was expanded to induce in vivo anti-tumor effects using the LMNPs@DOX. Wang et al. were also able to incorporate DOX-loaded mesoporous silica with PEGlyated LMNPs [13]. The PEGlylation process drastically decreases the surface tension of LMNPs and makes them even more transformable and suitable as drug carriers.

Xia et al. designed a polyethylene glycol (PEG) modified, zirconium dioxide (ZrO_2_)-coated LMNPs (LMNPs@pZrO_2_) as PTT reagents. LMNPs@ZrO_2_ infiltrates cancer cells and converts light energy into heat energy (Figure 6C) [58]. Wang et al. discovered the potential for LMNPs to undergo photothermal morphological changes in tumor cell endosomes where they are exposed to a slightly reduced pH [13]. A rapid increase of temperature was observed. More importantly, the LMNPs could be propelled by acoustic radiation force generated by an ultrasound field and undergo a shape transformation from spheres to rods. The same group also designed a leukocyte membrane-coated LMNPs that delivered anti-tumour drug aminopropyltrimethoxysilane (APTMS) and carbonylated β-cyclodextrin (β-CD) [31]. Fan et al. synthesized microspheres incorporating chitosan (CS), Ga liquid metal, and methotrexate (MTX) [59]. This design allowed anchoring of the anti-cancer drug upon the LM particle surface with a grafting molecule, both LM particles and MTX were encapsulated by chitosan, forming a spherical structure (Figure 6D). The shape transformation capability was also employed to suppress tumor growth. Table 2 summarizes different strategies developed for producing biofunctional LMNPs in treating cancer cells.

### 5.2. Medical Imaging

In addition to cancer therapy, biomedical applications of LMNPs in the field of medical imaging have been explored. Reports focusing on X-ray and photoacoustic imaging techniques are summarized below.

#### 5.2.1. X-ray Imaging

**Figure 7:**
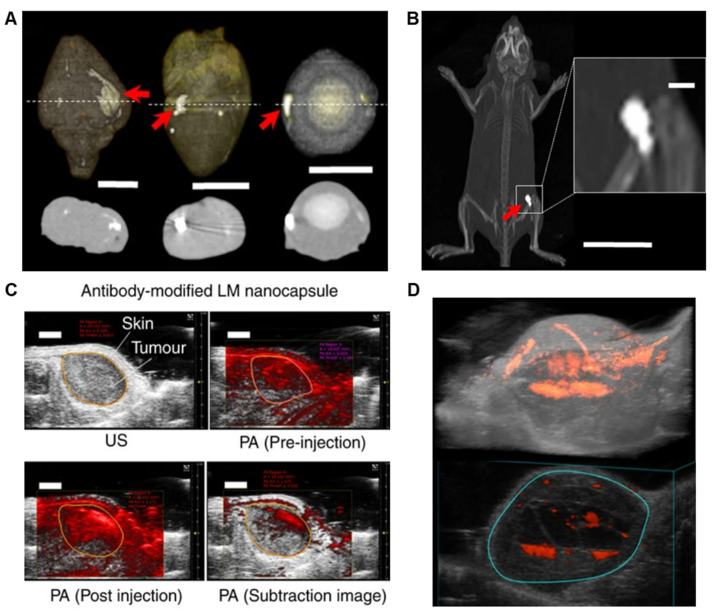
(**A**) X-ray images of cross-sectional views of rabbit heart, brain, and eyeball, respectively. The laser-irradiated sites are indicated by red arrows. Scale bar, 1 cm. (**B**) X-ray image of a living mouse with LMNPs injection. The laser-irradiated site is indicated by the red arrow. Scale bar, 3 cm (inlet: 2 mm). (**C**) LMNPs (100 μg/mL) were injected into the tumor site of living mice. Ultrasound (US) (grey) and PA (red) images were taken through the tumor by 750 nm laser excitation. The orange circle shows the part to be analyzed. Scale bars are 2 mm. (**D**) 3D imaging of the tumor site. The blue circle shows the tumor region. Reproduced with permission from NPG [7].

LMNPs used in vivo are not suitable to use as contrast agents because the contrast-concentration profile is not sufficient to warrant use. However, the X-ray contrast can be significantly enhanced by laser irradiation. LMNPs aggregate together under laser irradiation, inducing strong X-ray attenuation properties. Chechetka. et al. conducted in vivo experiment by injecting LM nanocapsules into animal organs (heart, brain, and eyeball from rabbits) and living mice, obtaining clear images upon laser irradiation (Figure 7A,B) [7].

#### 5.2.2. CT Imaging

Computed tomography (CT) imaging relies on computation to integrate X-ray images taken from different angles into cross-sectional, slice-like images. Zr is a classic CT contrast agent due to its high atomic mass. By coating LMNPs with ZrO_2_, the NPs can be used to generate diagnostic images. Xia’s et al. evaluated the effect of LM@pZrO_2_ on CT imaging, concluding that CT value is positively related to the concentration of the material [58]. This work also conducted in vivo experiments by injecting LM@pZrO_2_ NPs of different concentrations in tumor sites of mice, confirming that LM@pZrO_2_ NPs can be used as a safe and effective contrast agent. The CT intensity gradually increases in the first 6 h of intravenous injection and then decreases after it has reached its maximum value.

#### 5.2.3. Photoacoustic Imaging

Photoacoustic (PA) imaging is based on the photoacoustic effects, by which a convertor material transforms light energy into heat energy. The foreign heat induces transient, regional expansion and, as a result, wideband ultrasonic acoustic waves. The laser-induced heating and acoustic-explosion properties of LMNPs make them an ideal candidate to produce a photoacoustic signal. Chechetka et al. examined this possibility by conducting in vivo experiments in mice [7]. This work demonstrates that LMNPs exhibit photoacoustic signals upon absorption of light with a wavelength of 670–970 nm (Figure 7C). The strongest photoacoustic signal is presented when the wavelength is 680 nm. Like other materials that gives photoacoustic signals, there exists a positive, linear relationship between the concentration of LMNPs and the magnitude of photoacoustic signals. When combined with modeling techniques, the injected LMNPs could congruently facilitate the construction of 3D models and provide a clear insight into the tumor’s size and shape (Figure 7D).

### 5.3. Ion Channel Regulation

LMNPs are capable of generating ROS upon the absorption of NIR light. Chechetka et al., exploited photothermal behaviour to regulate ion channels [7]. Ion channels are membrane proteins that serve important functions such as transmembrane potential regulation. ROS interacts with ion channels by changing cross-membrane electrical potentials. Therefore, LMNPs have been hypothesized to control ion channel activities via ROS generation. This work conducted experiment on temperature-activated transient receptor potential cation channel subfamily V member 1 (TRPV1) taken from mouse neuroblastoma and rat neuron cells, which are known to overexpress these ion channels. The cells were marked with Fluo-8, and pre-incubated with LMNPs, and then irradiated with a NIR laser. Upon laser irradiation, a large amount of ROS was generated, opening ion channels and calcium accumulation was observed as a consequence. As laser irradiation is necessary to activate the ROS generation, LMNPs can not only effectively control ion channels but also be turned on or off as needed. Some noteworthy advantages of LMNP are that they absorb light with a wide range of wavelengths and show good thermal and chemical stability.

### 5.4. Pathogen Treatment

Efficiently deactivating pathogens in an environmentally friendly manner has become a worldwide issue. The traditional use of chemicals and biologics such as antibiotics has resulted in severe resistance issues in certain bacterial populations. LMNPs have properties that may prove beneficial for killing pathogens, exciting the interest of researchers in studies discussed below.

**Figure 8:**
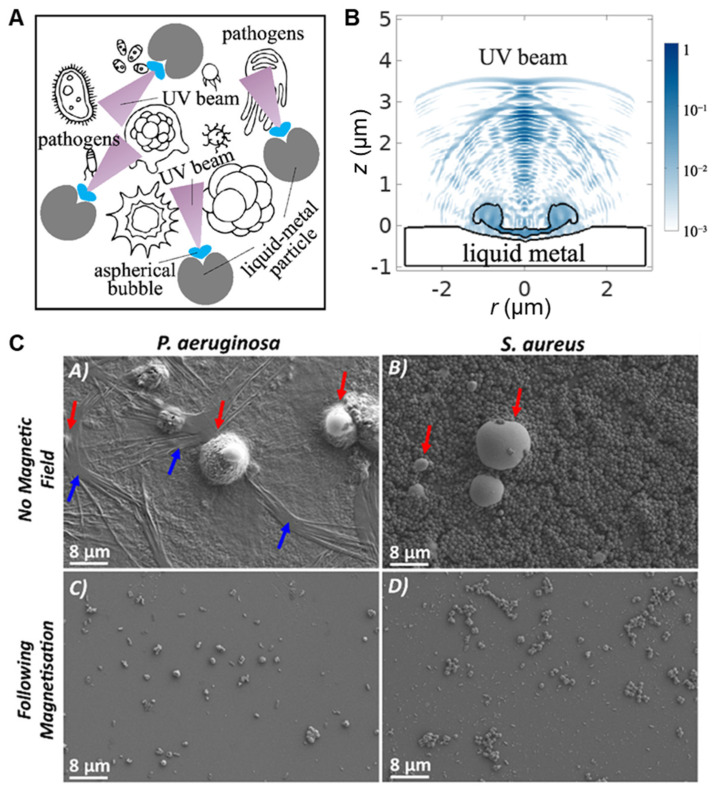
(**A**) Collapse of air bubbles in water near LMNPs for generating UV irradiation to kill pathogens. (**B**) Simulation results showing light emitted by an aspherical bubble near the liquid-metal surface. Reproduced with permission from NPG [60]. (**C**) SEM visualization of the *P. aeruginosa* and *S. aureus* biofilms following 24 h of growth with LMNPs (indicated by red arrows). Biofilms of these two bacteria groups and the self-produced extracellular polymeric substances are indicated with blue arrows. Applying an external rotating magnetic field for 90 min successfully removed the biofilms. Reproduced with permission from ACS [9].

UV-C light, typically with a wavelength of 200–280 nm, has been used as an environmentally friendly and highly efficient pathogen inactivation technique. Common pathogens, such as those responsible for cholera, polio, and certain bacterial or parasitic diseases, can be effectively eliminated by UV-C light. Unfortunately, if the initial UV dosage is not high enough, for instance, the percent inactivation is less than 99% or 99.9%, the surviving pathogens may recover. Boyd et al. proposed a concept of generating UV-C light on liquid metal microparticles (LMMPs) (Figure 8A) [60]. Based on the principle of sonoluminescence, the simulation results indicate the possibility of generating a UV light beam from an aspherical air bubble collapse near LMMPs (Figure 8B).

A significant complication and barrier to the eradication of pathogens is the existence of biofilms that provide a barrier to anti-pathogenic strategies. Biofilms can be categorized according to the type of organism forming the biofilm, such as bacterial or planktonic. Biofilms often grow upon biomedical devices and implants causing significant problems for infection control, requiring significant usage of antibiotics. Elbourne et al. proposed the use of liquid Galinstan-based LMNPs as a solution to the biofilm problem (Figure 8C) [9]. This work takes advantage of the morphological transformability of LMNPs to fight biofilms (*Pseudomonas aeruginosa* and *Staphylococcus aureus* in this experiment). When LMNPs are placed above a dynamic magnetic field (intensity of ~775 mGs), LMNPs move and spin at high speed and undergo shape-transformations from sphere to rod or “ninja stars” that could physically disrupt the biofilm matrix, and simultaneously lyse the cells. This approach has also been applied using zinc oxide, gold, silver, iron oxide, etc., suggesting a general strategy for biofilm control. Although no clear mechanism for the morphological transformation and anti-biofilm efficacy was explained in this work, we hypothesize that part of the anti-pathogenic effect and morphological transformation can be attributed to heating effects and resulting eddy current induced by an alternating or dynamic magnetic field.

### 5.5. A Glance of Biomedical Applications Using Bulk Liquid Metal

In addition to NPs, bulk and micro-sized liquid metal have also shown a vast potential in biomedical, chemical, and electronic applications, which have been reviewed elsewhere in detail [2,61,62,63]. Herein, we briefly discuss the bulk materials described previously for biomedical applications. In addition to LMNPs, Ga-based liquid metal alloys in bulk have been described in applications against cancer. EGaIn by itself can be used as a soft paste in PTT, powered by an alternating magnetic field (AMF) [11] or NIR light [64]. Incorporating magnesium metal into EGaIn, Wang et al. manufactured a soft metal paste that released heat upon NIR irradiation and studied its application in melanoma therapy [64]. GaIn-Cu paste has been used to enhance the effects of cryoablation. Hou et al. designed a PTT-cryoablation therapeutics specifically against melanoma, using Ga liquid metal as a platform [65]. Zhang et al. designed a drug delivery motor that incorporated a Ni cap and Al foil at the millimeter scale [66]. This motor could move freely within the body, and its direction could be controlled by external magnetic or electric fields. Using a liquid metal droplet robot with a hollow magnetic internal framework, Li et al. demonstrate the controlled release of DOX for targeted drug delivery and therapy trials to treat breast cancer cells (4T1) [67].

Ga-based liquid metals could also be applied to bioimaging. In bulk form, Ga metals can be used to generate clear CT images [7,68]. Wang et al. found out that clear CT image could be obtained and the duration of LM could also last up to 5 days [68]. In addition, liquid metal Ga has been used as a substitution for the traditional X-ray contrast agent, iohexol [69]. Liquid metal Ga produced high-resolution X-ray images that one could clearly identify the coronary network, while only the larger vessels could be observed in the case of iohexol. Moreover, the systematic biological evaluation of liquid metal also proved that it can be used for making biocompatible, functional, and implantable devices [23].

## 6. Conclusions and Outlook

Ga-based alloys possess a desirable combination of fluidity, electroconductivity, chemical reactivity, stimuli responsivity, and biocompatibility. Their ubiquitous oxide layer facilitates the mass production of micro- and nanoparticles at reasonable cost with equipment accessible to most researchers. However, there is still a large gap between the current state of LMNPs research and actual clinical applications in Food and Drug Administration (FDA) approved drugs or instruments. In comparison to conventional rigid NPs synthesized from materials such as gold, silver, iron oxide, rare-earth elements, etc., LMNPs still need to be proven in biomedical applications. There are many unresolved issues associated with the biomedical application of Ga-based alloys in both nano and bulk forms, and a guide to the future is given below.

*What are the mechanisms of anchoring?* It is accepted that the oxide skin of LMNPs is a versatile platform that can be used to anchor graft molecules. However, the precise chemistry of functionalization is still to be proven and therefore rigorous control has yet to be achieved. Whether the anchoring groups bind to the oxide layer or to the metallic surface underneath the oxide is still debatable. Investigating the surface binding using surface analytical techniques such as X-ray photoelectron spectroscopy (XPS) and time-of-flight secondary ion mass spectrometry (ToF-SIMS) is essential in future studies to provide mechanistic details and allow for full characterization.

*How could the production process be optimized to narrow down the size distribution?* In production methods such as sonication, the size distribution of LMNPs is related to the sonication time and power. However, with current top-down methods, the size distribution of LMNPs is typically broad. This limitation prevents researchers from applying LMNPs onto fields that have a strict and specific size requirement. We believe this issue could be resolved by introducing post-production processes such as centrifugation and filtration.

*How can the colloidal stability of LMNPs be enhanced?* Production strategies need to be optimized to enhance the stability of LMNPs in biological buffers. Current approaches rely on co-sonicating bulk liquid metals within a solution containing grafting macromolecules. However, almost no research has been conducted on evaluating the grafting density and the integrity of grafting macromolecules after the sonication process. The long-term colloidal stability of LMNPs in physiological environments is yet to be understood. Aggregation of NPs can directly or indirectly influence the cellular uptake and the toxicity profile, thereby leading to misrepresentative results and uncontrollable reproducibility of experiments [70]. Characterization techniques such as scattering (e.g., dynamic light scattering), optical spectroscopy (e.g., localized surface plasmon resonances), and microscopy (e.g., SEM and TEM) need to be used to in situ evaluate LMNPs’ physico-chemical properties and the colloidal stability in physiological fluids.

*What are the underlying mechanisms of Ga’s toxicity?* Since LMNPs may be applied to humans for disease treatment or medical imaging, a comprehensive understanding of its cytotoxicity is of top importance. There are a number of reports on the cytotoxicity of different forms of Ga. However, the underlying mechanism of toxicity (or otherwise) needs to be characterized. In order for this to be achieved, a thorough nanotoxicology study is required not just in vitro but using in vivo models using (for example) zebrafish and mice. If it can be determined that Ga^3+^ ions act like iron (III) ions and interfere with hemoglobin function, researchers would be better directed on where and how to apply LMNPs safely in the body. This is a significant limiting factor as things stand and therefore is an essential barrier to be surmounted before the field can progress.

*How does the oxide skin of LMNPs dissolve in the body?* The lowest pH value in physiological environments is ~5.5. However, the dissolution of Ga oxide generally requires extreme pH values (pH < 3 or pH > 11.5) at room temperature [52]. It is debatable how such an oxide can be dissolved in the tumor region, which has a typical pH value of ~5.5. Could higher body temperature facilitate dissolution processes? The mechanisms and processes need to be further explored. A thorough investigation of the stability of the oxide layer for LMNPs grafted with different anchoring groups needs to be conducted in physiological fluids at different temperatures and pH.

*What makes LMNPs absorb NIR light?* The photothermal conversion capability is essential in many applications of LMNPs. However, there are minimal reports describing the mechanism of LMNPs in photoconversions. No specific absorption peak near the NIR region can be detected for most reported LMNPs with or without functional coatings. Unlike NPs made from noble metals, Ga-based NPs exhibit surface plasmonic resonance peaks in the UV region [51,71,72].

*What makes LMNPs indispensable in biomedical applications?* Ga-based bulk alloys and NPs have properties that are attractive in biomedical applications, however, many other metals share similar properties with Ga and possess even superior qualities. For instance, Ga (electrical conductivity of 7.1 × 10^6^ S/m at 20 °C) is chemically less stable and less electroconductive than Au (4.1 × 10^7^ S/m) and Ag (6.3 × 10^7^ S/m). Additionally, it is not a metal of high density, which limits its utility in X-ray-based bioimaging. To this end, future studies need to focus on the shape flexibility of LMNPs, as this is their unique selling point. As shape has been found to be a key parameter in effective nanomedicine, this is probably an area worthy of more study [73,74].

## Figures and Tables

**Table 1:** Comparison of methods for the production of liquid metal nanoparticles (LMNPs).

Method	Size	Shape	Throughput	Costs ^1^
Sonication, e.g., [24,27,28,29] (Top-down)	10s to 100s nm (Polydisperse)	Mostly spherical	High	★★★
Liquid-based nebulization [30] (Top-down)	80 to 400 nm (Polydisperse)	Mostly spherical	Medium	★
Pressure-template [13,31] (Top-down)	>300 nm in diameter, >5 μm in length (Monodisperse)	Rod or needle-shaped	Low	★★
Shearing with a rotary tool [32] (Top-down)	6.4 nm to 10s μm (Polydisperse)	Mostly spherical	High	★★
Thermal decomposition [33] (Bottom-up)	12 to 46 nm (Monodisperse)	Spherical	High	★★★
Physical deposition [34] (Bottom-up)	25 to 100s nm (Polydisperse)	Spherical	Medium	★★★★

^1^ The number of asterisks (★) represents the cost of production; 1 means relatively low cost, while 4 means expensive.

**Table 2:** Strategies developed for producing biofunctional LMNPs for treating cancer cells.

Size and Shape	Surface Functionalization	References
Nanospheres, a diameter of ~107 nm	Grafting molecule: (2-hydroxypropyl)-b-cyclodextrin (designated MUA-CD) and thiolated hyaluronic acid (designated m-HA) Drug loaded: doxorubicin (DOX) Shape transformation: triggered by pH changes	[27]
Nanospheres, a diameter of ~150 nm	Grafting molecule: 1,2-distearoyl-*sn*-glycero-3-phosphoethanolamine-*N*-[amino(polyethylene glycol)-2,000] (DSPE-PEG_2000_-Amine) and 1,2-bis(10,12-tricosadiynoyl)-*sn*-glycero-3-phosphocholine (DC(8,9)PC) Shape transformation: triggered by NIR light	[7]
Nanospheres, hydrodynamic size of ~127 nm	Surface grafting: a surface mesoporous silica coating Drug loaded: DOX Shape transformation: N/A	[55]
~100 nm in diameter for LMNP@GOX and LMNPs	Grafting molecule: glucose oxidase (GOX) Drug loaded: N/A Shape transformation: triggered by NIR	[57]
Average diameters of GaNS, GaNR, and LMNR are 220, 255, and 237 nm, respectively	Grafting molecule: N/A Drug loaded: N/A Shape transformation: induced by ultrasound or laser	[12]
Nanospheres, a diameter of ~700 nm without silica coating; diameter of ~250 nm with a silica coating	Grafting molecule: Inorganic SiO_2_ shell using four silica sources (1) tetraethylorthosilicate (TEOS) (2) 3-mercaptopropyltriethoxysilane (MPTES), (3) bis(γ-triethoxysilylpropyl)-tetrasulfide (BTES) (4) tetraethylorthosilicate + bis(γ-triethoxysilylpropyl)-tetrasulfide (TEOS + BTES) Drug loaded: N/A Shape transformation: triggered by NIR	[54]
Nanorod with a diameter of 360–620 nm and a length of about 5.5 μm	Grafting molecule: N/A Drug loaded: N/A Shape transformation: triggered by NIR irradiation	[13]
Needle-like shape, length of ~7 μm and diameters of 800 ± 51 nm and 153 ± 37 nm at each end	Grafting molecule: a natural leukocyte membrane shell Drug loaded: aminopropyltrimethoxysilane (APTMS) and carbonylated β-cyclodextrin (β-CD) Shape transformation: trigger by NIR or pH changes	[31]
Nanospheres with GQD coating, a diameter of ~150 nm	Surface grafting: graphene quantum dots (GQDs) Drug loaded: DOX Shape transformation: trigger by light irradiation (wavelength = 635 nm)	[14]
Spherical particles, a diameter of 6–80 μm	Surface grafting: chitosan Drug loaded: methotrexate (MTX) Shape transformation: triggered by NIR	[59]
Non-coated nanospheres with a diameter of 120 ± 60 nmHydrodynamic size of ~120 nm for the LM@ZrO_2_ NPs	Surface grafting: ZrO_2_ and polyethylene glycol (PEG) Drug loaded: N/A Shape transformation: triggered by NIR	[58]
Nanospheres, a diameter of ~270 nm (LMNP@CM)	Surface grafting: polyethylene glycol and tumor cell membranes (CM) as antigens Drug loaded: N/AShape transformation: triggered by NIR	[56]
